# Systematic review of the effectiveness of community-based self-management interventions among primary care COPD patients

**DOI:** 10.1038/s41533-018-0111-9

**Published:** 2018-11-23

**Authors:** K. Jolly, M. S. Sidhu, E. Bates, S. Majothi, A. Sitch, S. Bayliss, H. J. Samuel Kim, R. E. Jordan

**Affiliations:** 1 Institute for Applied Health Research, University of Birmingham, Edgbaston, Birmingham B15 2TT UK; 2 School of Social Policy, University of Birmingham, Edgbaston, Birmingham B15 2TT UK; 3 St John’s College, University of Oxford, Oxford, OX1 3JP UK

## Abstract

COPD self-management reduces hospital admissions and improves health-related quality of life (HRQoL). However, whilst most patients are managed in primary care, the majority of self-management trials have recruited participants with more severe disease from secondary care. We report the findings of a systematic review of the effectiveness of community-based self-management interventions in primary care patients with COPD. We systematically searched eleven electronic databases and identified 12 eligible randomised controlled trials with seven included in meta-analyses for HRQoL, anxiety and depression. We report no difference in HRQoL at final follow-up (St George’s Respiratory Questionnaire total score −0.29; 95%CI −2.09, 1.51; *I*^2^ 0%), nor any difference in anxiety or depression. In conclusion, supported self-management interventions delivered in the community to patients from primary care do not appear to be effective. Further research is recommended to identify effective self-management interventions suitable for primary care populations, particularly those with milder disease.

## Chronic Lung Disease: Towards effective self-management in primary care

Further work is needed to ensure that self-management of chronic lung disease in primary care settings actively improves patients’ quality of life. While self-management is beneficial to patients in secondary care with severe chronic obstructive pulmonary disease (COPD), few studies have examined self-management effectiveness in primary care patients with milder COPD. Kate Jolly at the University of Birmingham, UK, and co-workers identified only 12 studies out of over 12,500 that specifically examined self-management in primary care. Of these, seven were suitable for metanalysis. The team found that community-based interventions to support self-management did not make a significant difference to patients’ perceived quality of life, or in reducing anxiety and depression. They call for further research to identify specific support that will help patients with mild to moderate COPD cope and adapt to the progressive condition.

## Introduction

COPD is a significant cause of morbidity and mortality; it accounts for a high consultation rate in general practice^1^ as well as significant hospital admission rates for exacerbations which drive the high cost of treatment.^2^ Support for self-management among people with COPD has been shown by systematic reviews to improve health-related quality of life (HRQoL) and reduce hospital admissions.^3^ However, most participants within self-management trials have been recruited from secondary care with more severe disease than is representative of the population of patients with COPD seen in general practice.^4,5^ In the UK and increasingly in many other countries, most people with COPD are managed in primary care. Deficiencies in self-management support have been identified in patients attending UK primary care with suboptimal rates of patients having a self-management plan, having received advice on diet or exercise or practical help to stop smoking in the previous year.^6^ Interventions to support self-management are heterogeneous and there is a lack of evidence as to whether effective interventions can be implemented and delivered in primary care settings. This is of increasing importance with calls to intervene to reduce risk in people with early stage COPD.^7^

There is a need for a systematic review to collate the evidence of the effectiveness of interventions to support self-management of patients with COPD identified from primary care with the intervention delivered in primary care or within a community setting. The aim of this review is to evaluate whether self-management interventions in COPD patients recruited from primary care lead to improved health-related quality of life, improved health outcomes and reduced health care utilisation.

## Results

### Study selection

**Fig. 1:**
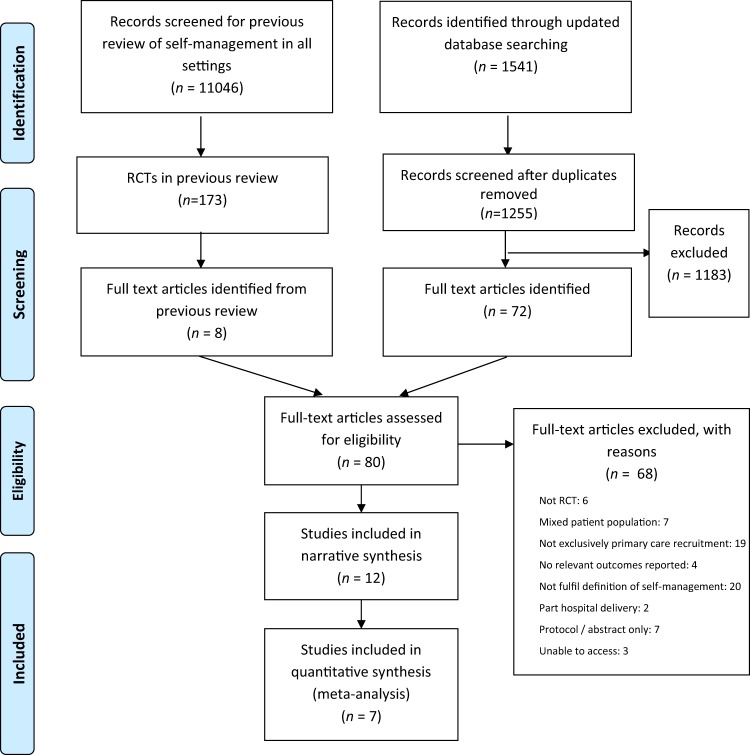
Article selection

The search results for the systematic review are shown in the PRISMA flow diagram (Fig. 1). The original search of databases from inception to 2012 identified 11,046 abstracts after removal of duplicates,^7^ from which eight full text articles were assessed. The updated search, focussed on primary care, identified 1255 abstracts from which 72 full text articles were assessed for eligibility; in total 12 articles met the full eligibility criteria. As outcome measures were heterogeneous between studies, only seven studies shared outcome measures which allowed inclusion in the meta-analyses.

### Overall study characteristics

Study characteristics of included studies are presented in Table 1 (for full details see Supplementary Table 1). Seven trials were individually randomised and 5 trials were cluster RCTs.^8–12^ Four trials were carried out in the UK, two in Australia and one each in The Netherlands, Sweden, Germany, US, China and New Zealand. Apart from one three-arm trial,^13^ all were two arm trials. Trials ranged from 52^14^ to 8217^9^ participants, including a total of 10,647 patients. The control arm of studies was most frequently usual care, with two studies providing information booklets as part of the control arm^15,16^ and one using usual care with an assessment of the patients’ health status every 2 months^9^. One study provided non-interventional brief telephone calls to usual care participants to control for the effect of the additional attention received by the intervention group.^11^ Duration of follow-up ranged from 3 months^17^ to 4 years.^9^ All participants were recruited from disease registers within general practices or community centres.

**Table 1:** Characteristics of included RCTs

Author year (RoB)	Setting	Intervention description	Intervention components^1^	Intervention deliverer	Intervention contacts	Intervention length	Sample size	Mean age (SD)	Sex (% male)	Mean FEV_1_% predicted (SD)	Maximum follow-up (months)	Outcome measures
Billington 2015 (low)	UK	Nurse-led telephone intervention	AP, E	Nurse practitioner	2	6w	73	Int: 72.1 (9.2) UC: 72.0 (11.0)	47.9		3	CAT; HSU; Exac.
Bischoff^13^ (low)	Netherlands	Modified version of Canadian “Living Well with COPD”	AP, BT, DK, E, Ex, HL, M, SA	Practice nurse	8-10	4–6w	165	Int: 65.5 (11.5) UC: 63.5 (10.3)	64.8	Int: 66.3 (16.5) UC: 63.5 (10.3)	24	CRQ; Exac; S-E
Coultas ^16^ (high)	USA	1. medical management; 2. collaborative management	DK, E	Nurse	1 + telephone	6 m	217	69 (8.2)	43.1		6	SGRQ; SF-36; II; HSU; S-E
Efraimsson ^14^ (high)	Sweden	Self–care education	BT, CP, DK, E, Ex, I, M, PS	Study nurse	2	12-20w	52	Int: 66 (9.4) UC: 67 (10.4)	50		5	SGRQ; Smoking
Freund ^8^ (low)	Germany	Care management intervention	AP, assessment, monitoring	Medical assistants	Mean 11	12 m	543 with COPD	Int: 71.6 (9.6) UC: 72.4 (9.6)	48.0	Median: Int 55.1 Con: 55.5	24	HSU; SF-12; EQ-5D Mort
Howard ^15^ (low)	UK	COPD breathless manual	B, DK, R	Health psychologist	3	5w	222	Int: 71.2 (10.4) UC: 73.2 (11.4)	42.5	Int: 55.9 (15.7) Con: 59.6 (15.9)	12	HSU; HADS; CRQ-SR
Lou ^9^ (high)	China	Management group	DK, Ex, I, M, PS, SC, V	GPs and specialists	48+	2 yrs	8217	Int: 61.6 (13.5) UC: 61.4 (13.2)	48.0		48	BODE; HADS; Smoking, HSU; Mort
Mitchell ^19^ (low)	UK	SPACE FOR COPD manual	AP, E, DK, Ex	Physiotherapist	3	6w	184	Int: 69 (8.0) UC: 69 (10.1)	54.9	Int: 56.0 (16.8) UC: 59.6 (17.4)	6	CRQ; HADS; ISWT; ESWT; S-E; Smoking
Rea ^10^ (low)	New Zealand	Chronic disease management programme	CP, HL, M	Nurse and respiratory physician	Unclear 16	Unclear 12 m	135	68	(42)	51.1	12	HSU; CRQ
Taylor ^18^ (high)	UK	Modified Expert Patients Programme	HCP, HL, M	Peers	7	7w	116	Int: 69.0 (9.8) UC: 70.5 (10.0)	45.7	Int: 53.9 (22.6) UC: 54.6 (23.4)	6	SGRQ; EQ5D; HRQoL HADS; S-E
Walters ^11^ (high)	Australia	Health Mentoring	DK, HCP, M, SE	Community health nurses	16	12 m	182	Int: 68.2 (7.9) UC: 67.3 (7.6)	52.7	Int: 54.0 (13.4) UC: 56.4 (13.2)	12	SF36; SGRQ; HADS; PIH; CES-D; HSU
Zwar ^12^ (high)	Australia	Individualised care plan	DK, E, Ex, M, SC	Nurse and GP	9	6 m	451	Int: 65.8 (10.3) UC: 64.4 (10.3)	47.9		12	SGRQ; SF12; FEV1; Smoking; HSU

*BODE* body mass index, airflow obstruction, dyspnea, exercise capacity, *CAT* COPD assessment tool, *con* control group, *CRQ* chronic respiratory questionnaire, *HSU* Health service use, *ESWT* endurance shuttle walking test, *Exac* exacerbations, *HADS* Hospital Anxiety and Depression Scale, *II* Illness intrusiveness, *ISWT* incremental shuttle walking test, *Int* intervention group, *m* months, *Mort* mortality, *PIH* Partners In Health Scale for self-management capacity, *RoB* risk of bias, *S-E* self-efficacy, *SF-12* short form-12, *SF-36* short form-36, *SGRQ* St George’s Respiratory Questionnaire, *UC* usual care group, *w* weeks, *yrs* years.

^1^**Components:** *AP* action planning, *B* managing breathlessness, *BT* breathing techniques, *CP* care plan, *DK* disease knowledge, *E* exacerbation management, *Ex* exercise, *HCP* interactions with health care providers, *HL* maintaining healthy lifestyle, *I* infection prevention, *M* medications, *PS* psychological counselling, *R* relaxation, *SA* managing stress and anxiety, *SC* smoking cessation, *V* vaccination counselling.

### Participant characteristics

The mean age of participants ranged from 61 to 73 years and 48.1% of the participants were male. Where reported, mean forced expiratory volume in one second (FEV1) ranged from 51^10^ to 66%^13^ of predicted values in the intervention arms at baseline. Apart from one study,^12^ the eligibility criteria of the studies was an FEV1/FVC < 0.7; other criteria included FEV1 < 80% predicted,^11,16,18^ MRC 3 or more,^15^ MRC 2 or more^19^ and at high risk of hospital admission.^8^

### Intervention characteristics

Interventions were heterogeneous and duration ranged from one month^15^ to at least 2 years.^9^ Interventions were delivered by a variety of health care professionals (GPs, nurse practitioners, medical assistants, respiratory physician nurses, health psychologists and trained peers); several studies used combinations of health professionals to deliver the intervention. Some studies used experienced health care professionals who were not specifically trained to deliver the intervention,^10,14,17,19^ in two studies the training was not specified,^13,18^ whilst most training of health care professionals ranged from 8 hours^16^ to 2 days.^8,9,12^ The content of the interventions varied from being focussed mainly on exacerbation management and responding to participants self-management queries^17^ to very comprehensive programmes including information about educational materials, physical activity advice, smoking cessation, breathing and medication management.^9,14,19^

### Outcome measures

Health-related quality of life (HRQoL) was reported by 10 studies, but only the St George’s Respiratory Questionnaire (SGRQ) and Chronic Respiratory Disease Questionnaire (CRQ) were included in more than one study allowing meta-analysis of these measures. Five studies included the SGRQ,^11,12,14,16,18^ but this was reported variably. Three studies included CRQ, reporting domains of dyspnea, emotions, fatigue and mastery.^13,15,19^ One study reported the CAT.^17^ Other measures included the Hospital Anxiety and Depression Scale (HADS),^9,11,15,18,19^ EQ-5D, exercise capacity, lung function, dyspnea and health care utilisation.

### Quality appraisal and risk of bias of included studies

**Fig. 2:**
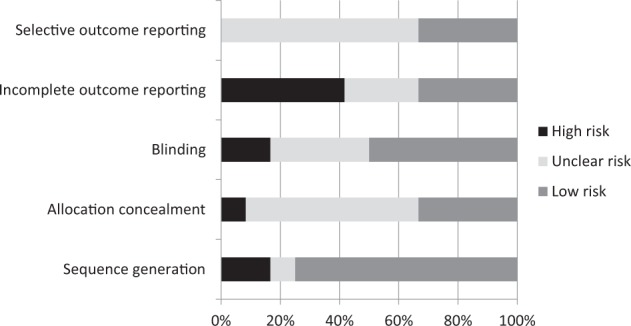
Quality assessment of included studies Percentages represent the percentage of included articles having a high risk of bias (black bar), unclear risk of bias (light grey bar) or low-risk (medium grey bar)

Figure 2 summarises the methodological quality and risk of bias for included studies, full details are given in Supplementary Table 2. Nine trials explicitly stated the process of randomisation. Details of allocation concealment and/or blinding were either poorly described or not provided. Four studies provided a complete description of drop outs and withdrawals, with significant information about the reasons for why participants dropped out or were lost to follow-up. However, four studies did not report an intention to treat analysis, ^9,11,16,18^ one had a baseline imbalance in lung function that was not adjusted for in the analysis,^12^ and one study did not report the method of randomisation.^14^

### Effectiveness results

**Fig. 3:**
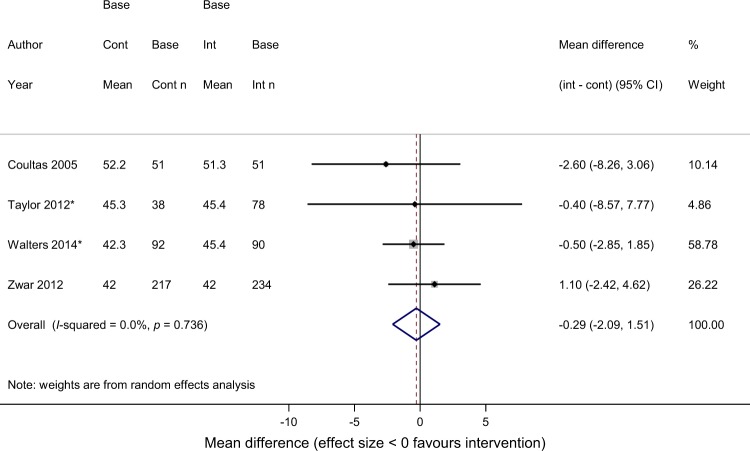
Meta-analysis of SGRQ-Total scores *adjusted results used

In the meta-analyses, there was no difference in HRQoL measured by the SGRQ at final follow-up (SGRQ total score −0.29, 95%CI −2.09, 1.51; *I*^2^ 0%) (Fig. 3), nor any significant differences in the SGRQ domains (Table 2 and supplementary figures 1-3). The SGRQ activity (mean difference −1.56, 95%CI −4.22, 1.10; *I*^2^ 0%), symptoms (mean difference −1.47, 95%CI −4.74, 1.80; *I*^2^ 0%) and impacts scores (mean difference −0.63, 95%CI −2.65, 1.38; *I*^2^ 0%) were in the direction favouring the self-management interventions, but did not reach statistical significance. No trials in the meta-analyses reported a statistically significant finding for the SGRQ total or any of its domains. Three of the trials in the meta-analysis did not report intention to treat analyses^11,16,18^ and one had baseline imbalance in lung function.^12^

**Table 2:** Effect of self-management interventions on outcomes: meta-analyses

Outcome	No. of studies	Studies	No. of participants	Summary mean difference (95% CIs)	*I*^2^ (%)
SGRQ-total	4	Coultas; Taylor; Walters; Zwar	851	−0.29 (−2.09, 1.51)	0.0
SGRQ-impacts	3	Coultas; Taylor; Walters	400	−0.63 (−2.65, 1.38)	0.0
SGRQ-symptoms	3	Coultas; Taylor; Walters	400	−1.47 (−4.74, 1.80)	0.0
SGRQ-activity	3	Coultas; Taylor; Walters	400	−1.56 (−4.22, 1.10)	0.0
CRQ-dyspnea	3	Bischoff; Howard; Mitchell	516	0.76 (−0.24, 1.76)	90.9
CRQ-emotions	3	Bischoff; Howard; Mitchell	516	0.85 (−0.20, 1.90)	93.5
CRQ-fatigue	3	Bischoff; Howard; Mitchell	516	0.45 (−0.26, 1.16)	83.3
CRQ-mastery	3	Bischoff; Howard; Mitchell	516	0.57 (−0.24, 1.38)	89.5
HADS anxiety	4	Howard; Mitchell; Taylor; Walters	676	−0.35 (−0.91, 0.21)	37.1
HADS depression	4	Howard; Mitchell; Taylor; Walters	676	−0.59 (−1.51, 0.33)	82.0

The meta-analysis results for the CRQ domains of dyspnea, emotions, fatigue and mastery were in the direction favouring the self-management interventions, but none reached statistical significance and there were high levels of heterogeneity (Table 2 and supplementary figures 4-7). Howard et al., reported statistically significant differences between intervention and control for all four CRQ domains for an intervention that used cognitive behavioural therapeutic approach to address dyspnea.^15^

Of the trials that were not included in the meta-analyses, Billington et al.^17^, which had a low risk of bias, reported a significant and clinically important difference^20^ between the COPD Assessment Test (CAT) scores of the intervention and control groups at 3 months after adjusting for baseline CAT scores (mean difference 2.38, 95% CI 4.40, 0.36; *p* = 0.02), but this did not remain significant after adjusting for age, smoking and FEV1% predicted at baseline. In contrast, Effraimsson et al. reported statistically significant differences in the change from baseline to follow-up at 5 months in the SGRQ domains of symptoms, activity and impacts between the intervention and usual care groups favouring self-management.^14^ Rea, for which risk of bias assessment was largely unclear, reported significantly greater improvements on the CRQ domains of fatigue and mastery compared to usual care, but no difference for dyspnea or emotional functions.^10^

For generic quality of life, Freund et al. reported significant improvement in quality of life (SF-12) in the intervention group compared with the usual care group at 24 months (differences 1.16, 95% CI 0.24, 2.08) on the physical component and 1.68 (95% CI 0.60, 2.77) on the mental component).^8^ General health (difference on EQ-5D 0.03, 95% CI, 0.00, 0.05) was also significantly improved at 24 months.^8^ In both trials reporting the SF-36, there were no differences for any of the SF-36 domains between intervention and control.^11,16^

In the meta-analyses, HADS anxiety and depression were not significantly different between intervention and controls; heterogeneity was substantial for the depression analysis and two of the included trials were at high risk of bias due to lack of use of intention to treat analysis.^11,18^ However, in Lou et al., which also did not undertake intention to treat analysis, significantly lower proportions of intervention participants had a score of 8 or more (indicating possible anxiety or depression) on the HADS anxiety and depression scales at 4 years follow-up.^9^

Seven of the trials reported health care utilisation outcomes with most reporting no statistically significant differences at follow-up in ED visits^12,15,16^ and hospital admissions.^8,10,12,16^ Billington et al. had too few emergency department (ED) visits to make a comparison;^17^ Freund, Coultas, Zwar, Howard and Rea et al. did not show statistically significant differences at follow-up in ED visits^12,15,16^ and hospital admissions.^8,10,12,16^ At 4 years follow-up, Lou et al. reported 16.1% (95% CI 9.3, 23.2) fewer all-cause hospital admissions and 18.1% (95% CI 10.2, 27.9) lower ED visits in the intervention compared to the usual care group.^9^

Distance walked in the 6-minute walk test was statistically significantly greater in the intervention than usual care group (43 m; 95%CI 12, 75) in Lou et al. at 4 years follow-up.^9^ Mitchell et al. reported a significant difference in duration of the endurance shuttle walk test at 6 weeks, sustained to 6 months; but the incremental shuttle walk distance did not reach the minimally important difference within the intervention group or between groups.^19^

Only two trials reported lung function.^9,12^ At 4 years follow-up there was a significant difference in lung function between the intervention and usual care groups in Lou et al., with the self-management group having a smaller reduction in FEV1%predicted.^9^ Zwar et al. reported no difference in FEV1% predicted between groups at 12 months follow-up.^12^

In Lou et al., after 4 years, the mean MMRC dyspnoea score in the self-management group decreased by a mean of 0.4 (SD 0.3), whereas in the control group, this score increased by 0.3 (SD 0.2); difference between the 2 groups *p* < 0.001.^9^

Only Bischoff reported exacerbations. Frequency of exacerbations did not differ between the groups, but compared with usual care, more exacerbations in the self-management group were managed with prednisolone, antibiotics, or both (difference 3.98, 95% CI 1.10, 15.58).^13^

Two trials reported mortality, Freund reported no difference, whereas Lou reported a significantly lower mortality rate in the SM group at 4 years follow-up (difference 9.0%, 95% CI 5.6, 12.7; *p* < 0.001).^9^

## Discussion

Despite extensive searches, we identified only 12 RCTs. In contrast to studies among more severe COPD patients, community-based interventions to support self-management for COPD in primary care were not found to be effective in meta-analyses overall in improving health-related quality of life or in reducing anxiety and depression. Although effects favoured the intervention groups, they were not statistically significant. However, despite the lack of statistical significance, the differences seen on the CRQ dyspnea, emotions and mastery scores were consistent with the minimally clinically important difference of 0.5, but this was driven largely by one trial which focused on dyspnea management.^15^ The heterogeneity of outcome measures precluded synthesis of the full evidence. In contrast to most of the other studies, one large trial undertaken in China,^9^ which could not be included in the meta-analyses, reported clinically and statistically significant improvements in mortality, HRQoL, anxiety and depression, dyspnea, exercise capacity, lung function and hospital ED visits and hospital admissions. The patient population was similar to that of the other included trials. The intervention was very intensive, involving education sessions every 2 weeks for 2 years, thus unlikely to be reproduced in other primary care settings; the intervention also included secondary care specialists reviewing the patients in the primary care setting every 2 months, and the usual care may well have been less comprehensive than that provided in some other countries, with participants having low levels of COPD medications and a high mortality in the usual care group.^9^ Additionally, the trial had differential follow-up between the intervention and control groups (81.4 vs 69.7%) and no intention to treat analysis.

Another large trial not included in the meta-analyses, but generally well conducted, Freund et al.,^8^ reported positive effects on HRQoL, but no effect on health care utilisation. This included patients with type II diabetes and heart failure as well as COPD and thus reported few characteristics specific to the COPD participants, so it is difficult to compare the participants to those of the other included trials. This intervention was also intensive, with a mean of 11 contacts, and included intervention components that were tailored to the individual patient through collaborative goal setting.

The findings of this review differ from those reported in the 2014 Cochrane review which reported a significant improvement in HRQoL in patients allocated to self-management support for COPD (SGRQ total 3.51, 95% CI 1.65, 5.37) and reduction in respiratory admissions (OR 0.57, 95% CI 0.43, 0.75) compared to usual care.^3^ The trials in the Cochrane review mainly recruited participants from secondary care settings, some of which only included participants with GOLD 3 or 4 or who had a recent hospital admission, and thus on average, the participants of the included trials had more severe disease than in our review. In line with other systematic reviews of self-management support in a range of settings, we did not find significant differences in anxiety or depression.^3,21^ An RCT published since our searches were undertaken, reported the findings of a telephone health coaching self-management intervention in a UK primary care population with only mildly symptomatic COPD.^22^ In keeping with the findings of our systematic review this trial also reported no difference between study groups for the SGRQ-total score (mean difference −1.3, 95%CI −3.6 to 0.9) or HADS at 12 months follow-up.^22^

We did not plan sub-group analyses to explore the effects of individual components of the self-management interventions. However, we note that only four of the interventions in our review included action plans as a component.^8,13,17,19^ This contrasts to the findings of the Cochrane systematic review ^3^ where sub-analyses on the use of action plans could not be performed because almost all the studies included COPD exacerbation action plans.

Whilst the number of published RCTs of COPD self-management support in primary care is limited there have been a range of other primary care based trials addressing the self-management support and care of patients with COPD alongside other long term conditions. Kennedy et al. trained practice staff taking a systems level approach, reporting no improvement in HRQoL;^23^ Cartwright^24^ and Steventon^25^ evaluated a telehealth intervention reporting a reduction in hospital admissions and on mortality, but also no effect on quality of life.

A recent qualitative systematic review of barriers and facilitators to self-management of COPD may offer potential explanations as to the lack of effect of the primary care COPD self-management interventions.^26^ This highlighted the importance of the emotional and psychological burden of COPD.^26^ This is supported by evidence that interventions that address mental health have been shown to be more effective than those focussing only on symptom management.^3,27^ Whilst some of the trials included in our review included the management of stress or anxiety, this was not universal. In addition, qualitative research with health care professionals who provide self-management support has identified the role of life circumstances impacting on people’s ability to engage in self-management.^28^ Practitioners’ speciality, experience and interest in COPD influences how COPD self-management is supported,^28^ which might account for smaller effects in COPD self-management delivered by primary care teams compared to specialists. In addition, the interventions targeted different self-management behaviours, thus some interventions might be expected to impact some of the outcomes more than others. Whilst only three of the trials overtly addressed anxiety and depression through relaxation and psychological interventions,^9,13,15^ most other trials included disease education, breathing management and exercise, which all try to address the cycle of activity leading to breathlessness which leads to anxiety. HRQoL is a broad concept, encompassing the impact of COPD on activities, psychological health and social functioning. The trial interventions addressed elements of different components of HRQoL and as 10 of the 12 included trials included HRQoL as an outcome measure, usually using a disease specific HRQoL measure, the authors clearly considered that the intervention components had the potential to impact HRQoL.

Our review has several strengths and some limitations. We completed an extensive review of published and grey literature and included evidence from 12 RCTs, although only seven contributed to the meta-analyses. The primary outcome of our review is HRQoL. Whilst this has been shown to be sensitive to self-management interventions in patients with more severe disease recruited from secondary care settings,^3^ or after hospital admission, it may be less sensitive to change in people with less severe symptoms managed in primary care. In addition, as people adapt to their disease their perception of their HRQoL may change. Our search strategy was comprehensive. However, RCTs varied considerably in sample size and nature of interventions. A wide range of different outcomes was reported and only one study reported exacerbations. Although we were able to make some comparisons across studies, there may be sources of heterogeneity where there are differences in study design or population characteristics. Further, there was missing information in the reporting of study methods and results which limit the application of our findings. Some trials reported a high loss to follow-up,^15^ imbalance in baseline characteristics,^12,14,18^ or baseline characteristics on only those who were followed-up.^16^ Five of the trials were cluster design, but none reported an intra-cluster correlation coefficient, so we were unable to adjust for clustering in the meta-analyses. Few studies reported the fidelity of delivery of the intervention, so descriptions of the intervention are those planned, rather than those received.

With the limited number of trials reporting each outcome measure it was not possible to assess publication bias.

A considerable challenge of undertaking evidence syntheses in COPD is the range of outcome measures commonly used, measuring different domains and therefore precluding synthesis by meta-analysis. This limits the numbers of participants in each analysis. Additionally, a lack of standardisation in relation to reporting health care utilisation and exacerbations limits comparison between studies. Future consensus among the respiratory research community on a standard reporting framework would be beneficial.

The heterogeneity of the different populations and interventions are a challenge to interpreting the findings in relation to the features of self-management interventions in primary care. A recent individual patient data (IPD) meta-analysis reported that self-management for COPD was more effective in patients with more severe airflow limitation, which would be in keeping with our findings of limited effectiveness.^29^ The IPD did not explore subgroups based on recruitment setting.^29^

In conclusion, our findings fail to show a significant benefit of supported self-management for primary care patients with COPD. This has global importance for practice at a time when many countries are moving to a case-finding approach based in primary care to identify COPD patients earlier. From these findings it is difficult to recommend self-management support delivered in primary or community settings for people managed in primary care unless they are of higher intensity, which may not be feasible in most usual primary care settings. In a resource limited environment greater health gains are likely from targeting self-management support to people who have had a recent hospital admission for an exacerbation or identified from secondary care, who are likely to be more limited by their symptoms. For patients willing to attend a centre-based group programme, pulmonary rehabilitation is an effective intervention.^30,31^ Self-management interventions overall may need greater personalisation, as not all people benefit from the current approaches,^32,33^ and our evidence suggests that these services should be delivered outside of usual primary care. However, whilst self-management support as an ‘intervention’ in its current form is not effective in people with mild/moderate COPD, people living with the condition may still value support. Further research is recommended to identify the support that will help people self-managing and adapting to life with mild/moderate COPD to reduce the impact of this slowly progressive condition.

## Methods

This was a protocol-driven systematic review registered on the PROSPERO register of systematic reviews (CRD42016043958).^7^ No ethical approval was required for this review as it used published data. The idea for the review arose from a previous systematic review undertaken by the authors which used a very broad definition of supported self-management interventions in all settings to try to unpick the most effective components of self-management interventions.^7^ This review is confined to primary care patients and uses a narrower definition of supported self-management.^3^

### Eligibility criteria

To be included, studies had to be randomised controlled trials.

For inclusion we required at least 90% of the population to have COPD (or results reported separately for COPD participants); trials had to have recruited adult participants (aged 18 years or more) exclusively from primary care. The criterion of at least 90% having COPD was pre-specified and was to allow studies that were predominantly people with COPD to be included.

To be eligible, the self-management intervention had to be delivered entirely within primary care with no secondary or tertiary care attendance. Self-management support was as defined by Zwerink et al.^3^ to be an iterative process (more than two contact moments over time), ideally comprising formulation of goals and provision of feedback. Interventions had to comprise at least two of the following elements (i) smoking cessation; (ii) self-recognition of symptoms and self-treatment of exacerbations; (iii) an exercise or physical activity component; (iv) advice about diet; (v) advice about medication; (vi) advice on coping with breathlessness. Interventions could be delivered verbally, by telephone or face-to-face, by written or audio-visual material (or a combination of the aforementioned). Interventions that solely consisted of participant education, those which included pulmonary rehabilitation (as in- or out-patient) or those which were community-based but purely exercise were excluded.

Comparator arms could be no intervention, usual care, a control group, a sham intervention or another self-management intervention.

The primary outcome measure was change in HRQoL as measured by a validated score (including but not limited to the St George’s Respiratory Questionnaire (SGRQ), Chronic Respiratory disease Questionnaire (CRQ) or COPD Assessment Test (CAT)) as measured at last follow-up. Secondary outcomes were health care utilisation, exacerbations, anxiety/depression, exercise capacity, lung function, dyspnea and mortality.

### Search strategy

The following databases were searched from date of inception, with no language restrictions, for citations with potential relevance: MEDLINE, MEDLINE in Process, EMBASE all via OVID, Cochrane Central Register of Controlled Trials (CENTRAL), Cochrane Library (Wiley) CDSR, DARE, NHSEED and HTA databases, Science Citation Index (ISI), PEDro, PsycINFO (OVID) and Cochrane Airways specialised register. We include the search strategy for one database (Supplementary methods: Search Strategy for MEDLINE). This was then adapted appropriately for the remaining databases.

Initial searches were carried out as part of a previous systematic review of self-management in any setting undertaken by Jolly et al.^7^ with an updated search (with additional primary care focussed search terms) from 2012 to September 2017 to more efficiently identify additional publications with recruitment from primary care. The reference lists of retrieved articles and relevant reviews were searched manually. We have presented our process of article selection in Fig. 1.

### Study selection and data extraction

Articles identified in the search for the original systematic review were selected as described in Jolly et al. 2016.^7^ Titles and abstracts identified in the updated search to September 2017 were screened for potential eligibility independently and in duplicate and full text articles then reviewed independently by two reviewers for inclusion (KJ, HJSK, MS). Discussion with a third reviewer resolved uncertainty regarding inclusion. Data were extracted by a single researcher (SM) and checked independently for accuracy by two reviewers (EB, MD). Reported mean difference estimates and 95% confidence intervals (CIs) calculated from an analysis of covariance were preferred, otherwise, mean differences reported from an analysis of change scores, an analysis of final scores or change value were used.

Authors were approached for additional outcome data where these were not reported in a way that enabled inclusion in the meta-analyses.

### Methodological quality assessment

The quality of the eligible studies was independently assessed by two researchers (EB, MS) using the Cochrane Risk of Bias tool.^34^ Criteria included randomisation, sequence generation, allocation concealment, blinding (by outcome), completeness of outcome data, selective outcome reporting and presence of other potential sources of bias (e.g. baseline imbalance or incomplete baseline data). Studies were judged as having a high risk of bias if one or more domains were assessed as being of high risk.

### Statistical analysis

Data were synthesised and reported narratively and in tables following PRISMA guidelines. For data presented narratively we extracted mean differences from baseline to final follow-up (95% confidence intervals). Due to the heterogeneity of the interventions, a random effects model was used for the meta-analyses.^35^ Meta-analysis was done in Stata versions 14 and 15. Statistical heterogeneity was assessed using the *I*^2^ statistic. All continuous data were presented using a mean difference with 95% confidence intervals (95% CIs). Testing for funnel plot asymmetry was not done due to there being <10 studies in each analysis.^33^

## Electronic supplementary material

Supplementary information

## Data Availability

The data generated or analysed during this study are mainly included in this published article (and its supplementary information files). Additional data are available from the corresponding author on reasonable request.

## Electronic supplementary material

**Supplementary information** accompanies the paper on the *npj Primary Care Respiratory Medicine* website (10.1038/s41533-018-0111-9).
