# Supplementary material for: Systematic review of the effectiveness of community-based self-management interventions among primary care COPD patients
Source: NPJ Prim Care Respir Med. 2018 Nov 23;28:44. doi: 10.1038/s41533-018-0111-9 (PMC6251904; doi:10.1038/s41533-018-0111-9)
Supplement: Supplementary file 1 — Supplementary information [file 41533_2018_111_MOESM1_ESM.pdf]

Supplementary Table 1.Characteristics of included RCTs

Author year (country)	Intervention		Control	Recruitment**	Inclusion Criteria	Sample size	Mean age (SD)	Sex (%male)	Mean FEV_1% predicted (SD)	Follow-up (mths)	Outcomes
	Description	Length (wks)									
Billington et al (2015)<br><br>UK	**Nurse-led telephone interventions**<br>Usual care plus nurse practitioner telephone calls at week 3 and 5 post baseline. Unscripted phone calls to improve self-management lasting for a maximum of 25 minutes each. Information in phone calls focused on using a self-management plan to manage symptoms and initiate emergency medication use.	6 weeks	**Usual care**	General practices using disease registers	On the COPD register (FEV1/FVC<0.7), able to speak and read English to give informed consent and complete questionnaire.	73	Int 72.09 (9.24)<br><br>UC 71.97 (11.04)	47.9%		3 months	COPD assessment tool (CAT)<br>Health service use (A&E and hospital admissions)<br>Exacerbations in the previous 3 months
Bischoff et al (2012)<br><br>Netherlands	**Self-management programme**<br>Modified version of Canadian SMP titled "Living Well with COPD" (excl. of exercise programme, but incl. action plan, COPD disease knowledge, respiratory drugs, breathing techniques, managing exacerbations, maintaining a healthy lifestyle, managing stress and anxiety, and home exercise. Action plans were personalised.	4-6	**Usual care**<br>Care from their general practitioner when there is an exacerbation of symptoms. Did not receive care from practice nurse.<br><br>**Routine Monitoring group**<br>Practice nurses completing scheduled routine monitoring visits in general practice on top of usual	Patients on COPD registers at 15 general practices invited for baseline assessment	Aged 35 years or more FEV1/FVC<0.7	165	SM 65.5 (11.5)<br><br>RMG 65.8 (8.3)<br><br>UC 63.5 (10.3)	64.8	SM 66.3 (16.5)<br><br>RMG 65.8 (8.3)<br><br>UC 63.5 (10.3)	6, 12, 18 and 24<br><br>Reported 6 and 24	**Primary outcomes**<br>HRQoL<br><br>**Secondary outcomes**<br>Chronic respiratory questionnaire<br>Frequency of exacerbations and management<br>Self-Efficacy

Author year (country)	Intervention		Control	Recruitment**	Inclusion Criteria	Sample size	Mean age (SD)	Sex (%male)	Mean FEV_1% predicted (SD)	Follow-up (mths)	Outcomes
	Description	Length (wks)									
	Programme delivered by the practice nurse individually in 2 to 4 one hour sessions delivered in general practice. Each patient received minimum 2 sessions and 6 reinforcement tel. calls.		care. Assessment of symptoms, medication/inhaler adherence, frequency of exacerbations.								
Coultas et al (2005)<br><br>USA	Three arm trial: usual care (UC), nurse assisted medical management (MM); nurse assisted collaborative management (CM)<br><br>MM and CM delivered by nurses, MM designed to enhance patient knowledge about COPD and symptoms. CM was enhanced MM focusing on behavioural change. 1 x home nurse visit; X x telephone contact	6 months	**Usual care** received two educational booklets from the American Lung Association and advised to follow advice from physician.	17 sites as part of a urban academic health centre	Aged 45 years or more, current or former smoker with 20 pack year history, encountered cough, shortness of breath or wheeze during the past 12 months, FEV1/FVC<0.7 FEV1<80% predicted	217	69 (8.2)	43.1%		6 months	SGRQ<br>SF-36<br>Illness intrusiveness<br>Health care utilisation<br>Self-efficacy
Efraimsson et al (2008)<br><br>Sweden	Patients completed 2 visits with study nurse.<br><br>Intervention group completed 2 additional visits for self-care education<br><br>**Self –care education**	12-20	**No treatment.** Patients offered self-care education post data collection.	Referral by physician	Diagnosed with mild, moderate, severe or very serve COPD based on spirometry using the GOLD criteria	52	Int 66 (9.4)<br><br>Control 67 (10.4)	50%		Post intervention (5 month)	QoL<br>SGRQ<br>Smoking<br>Knowledge of COPD

**Author year (country)**	**Intervention**		**Control**	**Recruitment****	**Inclusion Criteria**	**Sample size**	**Mean age (SD)**	**Sex (%male)**	**Mean FEV_1% predicted (SD)**	**Follow-up (mths)**	**Outcomes**
	**Description**	**Length (wks)**									
	Based on motivational dialogue and tailored to severity based on the following component: description of the physiological effects of COPD, respiratory function, explanation of medication, prevention of exacerbations, assessment of breathing techniques, information about exercise, diet, pyscho-social counselling, infection prevention, and an individual treatment plan.<br><br>Nurse led intervention										
Freund et al (2016)<br><br>Germany	**Care management intervention** Protocol-based care management, including structured assessment, action planning, and monitoring delivered by medical assistants, compared with usual care	12 months	**Usual care**	General practices	Aged 18 years or over, receiving medical treatment for at least one of the following: T2DM, COPD, or chronic heart failure. Further, patients had to have a high risk for future hospitalisation.	543 with COPD (2076 overall)	Int 71.6 (9.6)<br><br>UC 72.4 (9.6)	48.0%		12, 24 months	Hospitalisations<br>QoL (SF-12)<br>EQ-5D<br>Mortality

Author year (country)	Intervention		Control	Recruitment**	Inclusion Criteria	Sample size	Mean age (SD)	Sex (%male)	Mean FEV_1% predicted (SD)	Follow-up (mths)	Outcomes
	**Description**	**Length (wks)**									
Howard et al (2014)	**COPD breathless manual (CM)**<br>Developed by health psychologist, consisting of a five week intervention divided into six sections covering: what is COPD, breathlessness, control breathlessness, exercises, relaxation, and summary. Aim was to manage distress to prevent A&E and hospital admissions. Patients asked to complete weekly exercises, provide a breathlessness rating, and use a relaxation CD.	5 weeks	**Information booklets**<br>Patients received a series of British Lung Foundation COPD booklets and were encouraged to work through them over 5 weeks.	General practices	Diagnosis of COPD (FEV1/FVC<0.7), or if FEV1 is equal to or above 80% predicted normal with other respiratory symptoms being present such as breathlessness or cough); (Medical research Council) MRC grade 3 or above; willingness to participate; informed consent; and read and write in English	222	Int 71.2 (10.4)<br><br>Control 73.2 (11.4)	42.5%	Int 55.9 (15.7)<br><br>Control 59.6 (15.9)	6 weeks, 6 months, 12 months	Hospital admissions (A&E and frequency and duration of admissions)<br>HADS<br>CRQ-SR
Lou (2015)<br><br>China	**Management group**<br>136 GPs undertook 2 days training.<br><br>48 lectures at 2-weekly intervals covering COPD information, observation of inhaler technique, medication adjustment, smoking cessation	Unclear – lectures for 2 years	**Usual care plus telephone** call to caregiver every 2 months to assess patient's condition.	14 community health care centres	COPD according to GOLD criteria. Excluded acute respiratory illness, other lung conditions or procedures that could	8217	Int 61.6 (13.5)<br><br>UC 61.4 (13.2)	48.0%		48 months	**Primary outcome:**<br>BODE (6MWD, FEV1%pred, MMRC dyspnoea scale, BMI)

Author year (country)	Intervention		Control	Recruitment**	Inclusion Criteria	Sample size	Mean age (SD)	Sex (%male)	Mean FEV_1% predicted (SD)	Follow-up (mths)	Outcomes
	Description	Length (wks)									
	counselling, vaccination information, encouragement to exercise, hand hygiene, rehabilitation and psychological counselling. Face-to-face contact with GP every 2 weeks. Team of specialists monitored GP reports and provided feedback.				affect spirometry.						**Secondary outcomes:** HADS Smoking, Health service use (hospital admissions, ED visits) Mortality
Mitchell et al (2014)<br><br>UK	**SPACE FOR COPD** Based on a manual containing educational material and home exercise programme. Includes goal setting, coping planning and case studies. Exercise regime included daily walking, resistance training using free weights 3 times per week. Action plan for exacerbation management included.<br><br>Delivered by a physiotherapist (30-45 min baseline consultation, two telephone calls)	6 weeks	**Usual care**	30 primary care practice registers	FEV1/FVC<0.7 MRC grade 2-5 Clinically stable for 4 weeks	184	SPACE 69 (8.0)<br><br>UC 69 (10.1)	54.9%	SPACE 56.04 (16.76)<br><br>UC 59.60 (17.42)	6 months	**Primary outcome** CRQ-SR dyspnoea<br><br>**Secondary outcomes** fatigue, emotion and mastery domains of the CRQ-SR, BCKQ, HADS, ISWT, ESWT, PRAISE, Smoking

Author year (country)	Intervention		Control	Recruitment**	Inclusion Criteria	Sample size	Mean age (SD)	Sex (%male)	Mean FEV_1% predicted (SD)	Follow-up (mths)	Outcomes
	Description	Length (wks)									
Rea et al (2003)<br><br>New Zealand	**Chronic disease management programme**<br>Care plan for regular maintenance checks and goals for lifestyle changes.<br><br>Education about smoking cessation, medication and use of inhalers<br><br>Delivered by respiratory physician (quarterly) and nurse (monthly). Single home visit.	12 months?	**Conventional care**<br>Access to guidelines and pulmonary rehabilitation	GP practices		135	68	(42)	51.1	12	Hospital admissions<br>Quality of life<br>Medication
Taylor et al (2012)<br><br>UK	**Better Living with Long term Airways disease (BELLA)**<br>Modified version of CDSMP used by the Expert Patients Programme (EPP). Addressed five core areas: defining the problem, decision making, finding and using resources, forming partnerships with health care providers, and taking action.	7 weeks	**Usual care**<br>Standard GP and respiratory care	10 primary care teams from disease registers	Aged 35 years or more, FEV1/FVC<0.7, post-bronchodilator FEV1<80% predicted or exacerbation of COPD leading to unscheduled health care	116	Int<br>69<br>(9.8)<br><br>UC<br>70.5<br>(10.0)	45.7%	Int<br>53.9<br>(22.6)<br><br>UC<br>54.6<br>(23.4)	2 (Post intervention) and 6 months	SGRQ<br>EQ5D<br>HRQoL<br>HADS<br>Stanford Self-Efficacy scales<br>Stanford Self-management scales

Author year (country)	Intervention		Control	Recruitment**	Inclusion Criteria	Sample size	Mean age (SD)	Sex (%male)	Mean FEV_1% predicted (SD)	Follow-up (mths)	Outcomes
	Description	Length (wks)									
	Delivered by two trained peers (one with COPD), using a structured, manualised, 3 hour session, weekly in a community setting										
Walters et al (2013)<br><br>Australia	**Health Mentoring**<br>Five core components; psychoeducation about COPD diagnosis and treatment; self-management skills; cognitive coping skills; communication skills; promoting self-efficacy.<br><br>Delivered by trained community health nurses giving sixteen 30 minute telephone consultations	12 months	**Usual care**<br>Group received GP care plus non-interventional brief phone calls.	Three divisions of general practice in Tasmania	Diagnosis of COPD, aged 45 years or more, seen by GP in past 12 months, > 10 year pack history, FEV1/FVC<0.7, FEV1 30-80% predicted	182	Int<br>68.2<br>(7.9)<br><br>UC<br>67.3<br>(7.6)	52.7%	Int<br>54.0<br>(13.4)<br><br>UC<br>56.4<br>(13.2)	6 and 12 months	SF36<br>SGRQ<br>Partners In Health (PIH)<br>Scale for self-management capacity<br>HADS<br>CES-D<br>Post-Traumatic Stress Disorder Checklist<br>Satisfaction with life and hospital admissions
Zwar et al (2012)<br><br>Australia	**Individualised care plan**<br>Trained nurses (COPD management) in partnership with GPs and other care providers.<br><br>Patients received two home visits and five telephone contacts with a nurse, and two consultations with their	6 months	**Usual care**	General practices using disease registers	Aged between 40-80 years, prescribed medications used for COPD, and seen the GP in previous 12 months.	451	Int<br>65.8<br>(10.3)<br><br>UC<br>64.4<br>(10.3)	47.9%		6 and 12 months	SGRQ<br>SF12<br>Lung function (FEV1)<br>Smoking<br>immunisation status for influenza and pneumococcus<br>Attendance at pulmonary

Author year (country)	Intervention		Control	Recruitment**	Inclusion Criteria	Sample size	Mean age (SD)	Sex (%male)	Mean FEV_1% predicted (SD)	Follow-up (mths)	Outcomes
	Description	Length (wks)									
	GP. Monthly contacts over the phone between GP and nurse along with two face to face meetings to discuss patient care.										rehabilitation<br>Patient knowledge of COPD<br>Health service use (GP and Hospital admissions)

Supplementary Table 2. Risk of bias

Author year	Sequence generation	Allocation concealment	Blinding	Incomplete outcome reporting	Selective outcome reporting	Other Risk of Bias
Billington et al (2015)	**Low**<br><br>"randomly assigned by a statistician not conducting the analysis, using a random number generator function on spreadsheet software"	**Unclear**<br><br>No details given about allocation concealment	**Low**<br><br>Participants not blinded; baseline data collected by self-completion questionnaire, administered and collected by a staff member not conducting the intervention.	**Low**<br><br>Follow-up at 3m<br>INT: 34/35<br>UC: 37/38<br>Lost to follow-up<br>1 patient died from each group	**Unclear**<br><br>Protocol not published	**Unclear**<br><br>More smokers in the intervention arm
Bischoff et al (2012)	**Low**<br><br>"We randomised participants by using a computer generated two block randomisation procedure"	**Unclear**<br><br>No details given about allocation concealment	**Low**<br><br>"To ensure that the investigators were blinded to individual treatment allocation, practice nurses informed the patients of their allocation."	**Low**<br><br>SM: 49/55 F/U at 24 M<br>RM: 46/55 F/U at 24 M<br>UC: 44/55 F/U at 24 M<br><br>16% dropout –"NO significant difference between dropouts and those completing trial"<br><br>ITT analysis of HRQoL OUTCOMES ONLY	**Low**<br><br>Published protocol<br>All outcomes reported	**Unclear**<br><br>Potential for cross contamination between treatment groups, but took steps to minimise this and checked for it at the end of the study
Coultas et al (2005)	**Low**<br><br>"Patients were randomly assigned to one of three intervention groups … using a computer-generated random list."	**Low**<br><br>Allocation concealment not described but person recruiting not aware of allocation at time of recruitment/ baseline data collected	**Unclear**	**High**<br><br>Not ITT analysis.<br>69.6% f/u balanced across groups - BUT Dropouts – more severe obstruction, higher distress, lower QoL	**Unclear**<br><br>Protocol not published	**Unclear**<br><br>full-Baseline characteristics published only for trial and f/u completers<br><br>Those invited but declining to participate were younger and more likely to be BME than participants

Author year	Sequence generation	Allocation concealment	Blinding	Incomplete outcome reporting	Selective outcome reporting	Other Risk of Bias
Efraimsson et al (2008)	**High**<br><br>"The randomization was performed when two patients with the same variables agreed to participate in the study by assigning each individual an identity number."	**Unclear**<br><br>**Limited information on lot drawing**<br>"An independent person drew lots for allocation to either intervention or control group."	**Unclear**<br><br>No information about blinding.	**High**<br><br>ITT analysis<br>significant information about drop-outs -BUT more smokers (30 vs 58%) and more "moderate COPD" (60% vs 37%) in drop out group	**Unclear**<br><br>No published protocol	**Unclear**<br><br>Only limited baseline data reported but patients matched for variables and statement "comorbidities similar"
Freund et al (2016)	**Low**<br><br>Primary care practices were randomly allocated to care management or usual care in a 1:1 ratio by block randomization with variable block lengths….. stratified randomization according to the population density of the participating practice sites.<br>We used computer-generated randomization lists (SAS, version 9.2 [SAS Institute]). A research assistant who was not otherwise involved in the project performed the central randomization.	**Unclear**<br><br>We informed physicians about their allocation via an official letter and asked them to inform participating patients.	**Low**<br><br>Blinding primary care physicians, medical assistants, and patients was not possible.<br>Blinded assessment of the primary and secondary end points as well as the responsible statistician to study group allocation.	**Low**<br><br>ITT analysis<br><br>**Int: /1093** F/U 12 M<br>**UC: /983** F/U 12 M<br><br>90.3% F/U at 12 M<br>92.4% F/U at 24m	**Low**<br><br>published protocol	**High**<br><br>No smoking status in baseline characteristics<br><br>No baseline lung function or description of diagnosis of COPD by spirometry<br><br>Enrolled patients were younger than those who declined to participate and had fewer all-cause hospitalizations<br>in the year preceding the trial than those who were not enrolled

Author year	Sequence generation	Allocation concealment	Blinding	Incomplete outcome reporting	Selective outcome reporting	Other Risk of Bias
Howard et al (2014)	**Low**<br><br>Simple blocked random sampling was undertaken.<br>Computerised random blocks of six at a time were randomised, three in each group.	**Unclear**<br><br>No details given about allocation concealment	**Unclear**<br><br>Participants were blind to group allocation.<br>Primary and secondary care staff were aware of patients' participation in the trial, but were unaware of group allocation.	**Low**<br><br>ITT analysis<br><br>45.5% loss to F/U at 6m<br><br>12M F/U:<br>CM: 97/112<br>IB: 102/110	**Unclear**<br><br>Protocol not published	**Low**
Lou et al (2015)	**High**<br><br>Centres with experience and those<br>without were then randomly allocated separately into the health management and control groups to prevent an imbalance<br>between interventions in centres with previous experience.	**Unclear**<br><br>No details	**Unclear**<br><br>No information on Blinding<br>-<br>Individual who conducted data collection not stated	**High**<br><br>No ITT analysis<br><br>Int: 3418/4197 at 4 year FU (81.4%)<br>UC: 2803/4020 at 4 year FU (69.7%)<br><br>Deaths:<br>Int: 14.5%; UC 19.4%	**Unclear**<br><br>No published protocol	**Unclear**<br><br>High number of deaths in the usual care arm
Mitchell et al (2014)	**Low**<br><br>"Participants were assigned to either usual care or SPACE FOR COPD via a web-based, concealed allocation programme, using simple randomisation codes prepared by the trial statistician"	**Low**<br><br>"via a web-based, concealed allocation programme,	**Low**<br><br>"The assessments at 6W and 6M were conducted by a member of the research team who was blind to randomisation allocation"	**Low**<br><br>ITT analysis at 6M<br><br>F/U at 6m:<br>SPACE: 65/89 (73.0%)<br>UC: 79/95 (83.2%)<br>"characteristics of dropouts and completers similar" but no detail.	**Low**<br><br>published protocol	**Unclear**<br><br>wide range of characteristics reported for all participants *BUT* gender imbalance at baseline (60%/ 49% male SPACE/ UC, greater smoking exposure in Intervention group

Author year	Sequence generation	Allocation concealment	Blinding	Incomplete outcome reporting	Selective outcome reporting	Other Risk of Bias
Rea et al (2003)	**Low**<br><br>“Fifty-one eligible practices with 116 GPs were randomized, using a set of computer-generated random numbers”	**Unclear**<br><br>no detail given on allocation concealment/ not explicitly stated	**Unclear**<br><br>No information on Blinding<br><br>Individual who conducted data collection not stated	**Low**<br><br>F/U at 12 M:<br>Int: 71/83 (85.5%)<br>Con: 46/52 (88.5%)<br><br>Significant information about drop-outs but no details of characteristics<br><br>ITT analysis for admissions BUT NOT FOR QoL	**Unclear**<br><br>no published protocol but outcome measures listed in paper reported	**Unclear**<br><br>Baseline data NOT fully reported<br>Smoking not included as a variable But statement “no significant difference in baseline demographic and clinical characteristics “
Taylor et al (2012)	**Unclear**<br><br>“Following baseline assessment, patients were randomised 2:1”	**Unclear**<br><br>“… patients were randomised 2:1, intervention: control, maintaining allocation concealment.”	**High**<br><br>“Questionnaires were self-completed by patients at home, in the presence of a researcher not associated with the intervention.”<br><br>Healthcare utilisation – **Low:** From medical records	**High**<br><br>F/U at 6 M:<br>Int: 61/78 (78.2%)<br>UC: 30/38 (78.9%)<br><br>Significant information about drop-outs But nil comment on baseline characteristics for dropouts vs completers.<br><br>Not ITT	**Unclear**<br><br>No published protocol	**High**<br><br>Baseline imbalance – data reported for all participants at baseline BUT imbalance in male: female ratio, current smoking and previous pulmonary rehabiltation between control and Intervention groups

Author year	Sequence generation	Allocation concealment	Blinding	Incomplete outcome reporting	Selective outcome reporting	Other Risk of Bias
Walters et al (2013)	**Low**<br><br>“After recruitment, practices were randomised using a code generated by investigators from a random numbers table stratified in blocks of four”	**Low**<br><br>“Allocation occurred independently using sequentially numbered, opaque and sealed envelopes.”	**Low**<br><br>Intervention vs sham intervention<br><br>“Blinding of participants or research officers was not possible given the nature of the study.”	**High**<br><br>F/U at 12 M:<br>Int: 74/90 (82.2%)<br>UC: 80/92 (87.0%)<br><br>Significant information about drop-outs , BUT no comparison of characteristics of dropouts vs trial completers<br><br>Not ITT analysis	**Unclear**<br><br>No published protocol	**High**<br><br>Baseline characteristics reported for all participants<br>Baseline imbalance in Smoking exposure (43.4 vs 53.9 pack years), current smoking 36 vs 48 % and medication.<br><br>Reported low fidelity of delivery of the intervention
Zwar et al (2012)	**Low**<br><br>“A cluster randomised trial, with randomisation at the level of the practice, was conducted to avoid contamination between intervention and control groups.”<br><br>“A researcher who took no further part in the study randomised practices”<br><br>Protocol- computer generated list and sealed envelopes	**Low**<br><br>A researcher who took no further part in the study randomised practices to intervention or control groups, with allocation concealment.	**Low**<br><br>Assessments were conducted by project officers who took no part in the intervention and were blind to group allocation.	**Unclear**<br><br>ITT analysis<br><br>330/451 (73.2%) completed 12 M F/U	**Low**<br><br>published protocol	**High**<br><br>Baseline imbalance in lung function

Supplementary Figure 1: Meta-analysis of SGRQ-impacts scores

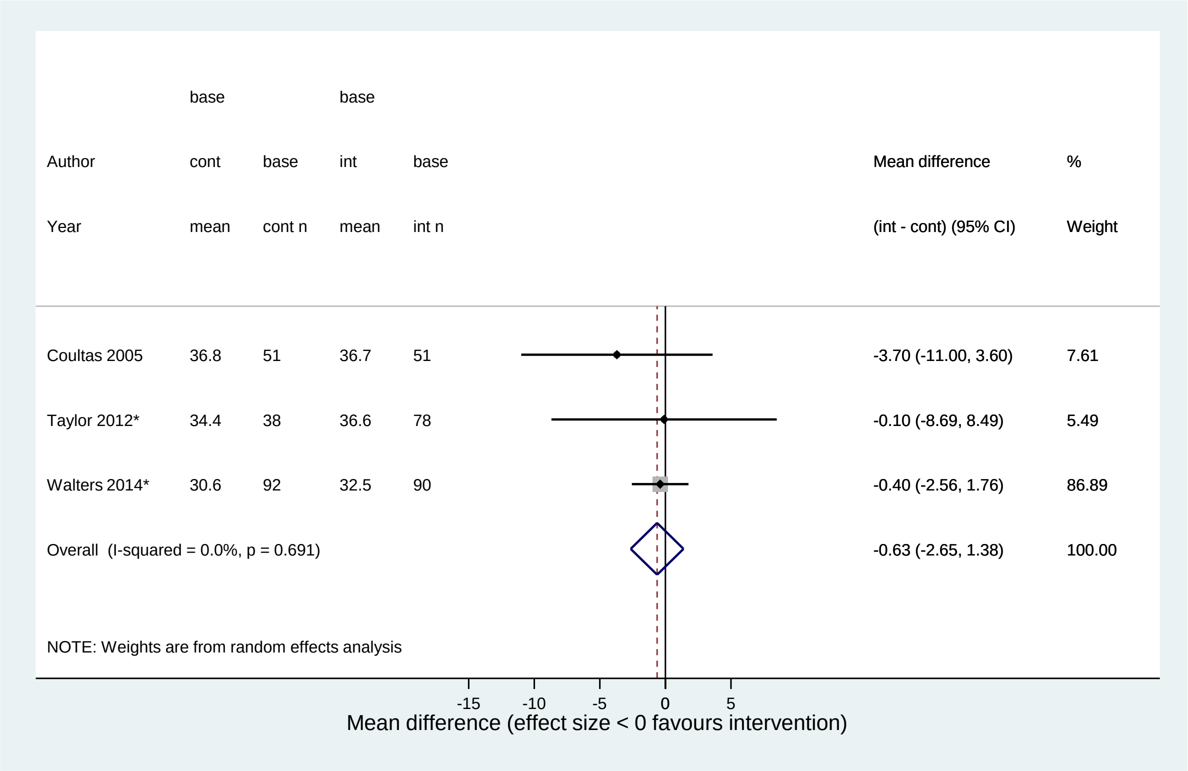

Supplementary Figure 2: Meta-analysis of SGRQ-symptoms scores

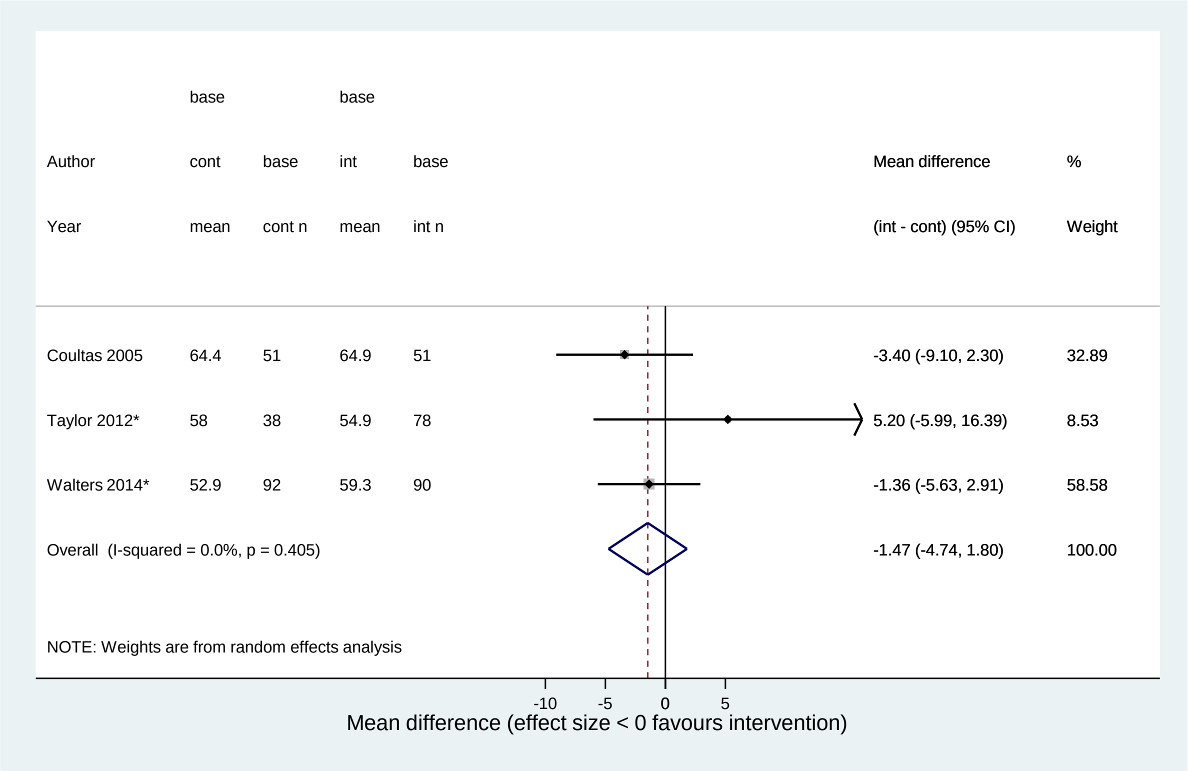

Supplementary Figure 3: Meta-analysis of SGRQ-activity scores

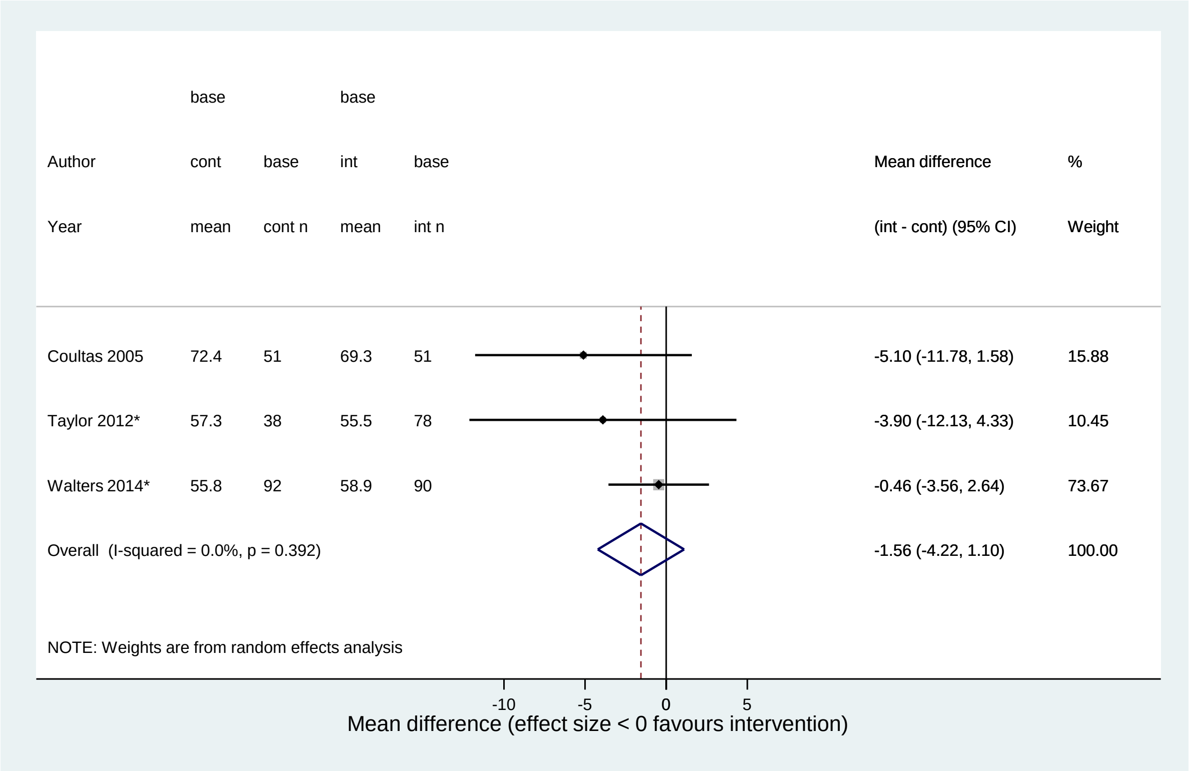

Supplementary Figure 4: Meta-analysis of CRQ-dyspnea scores

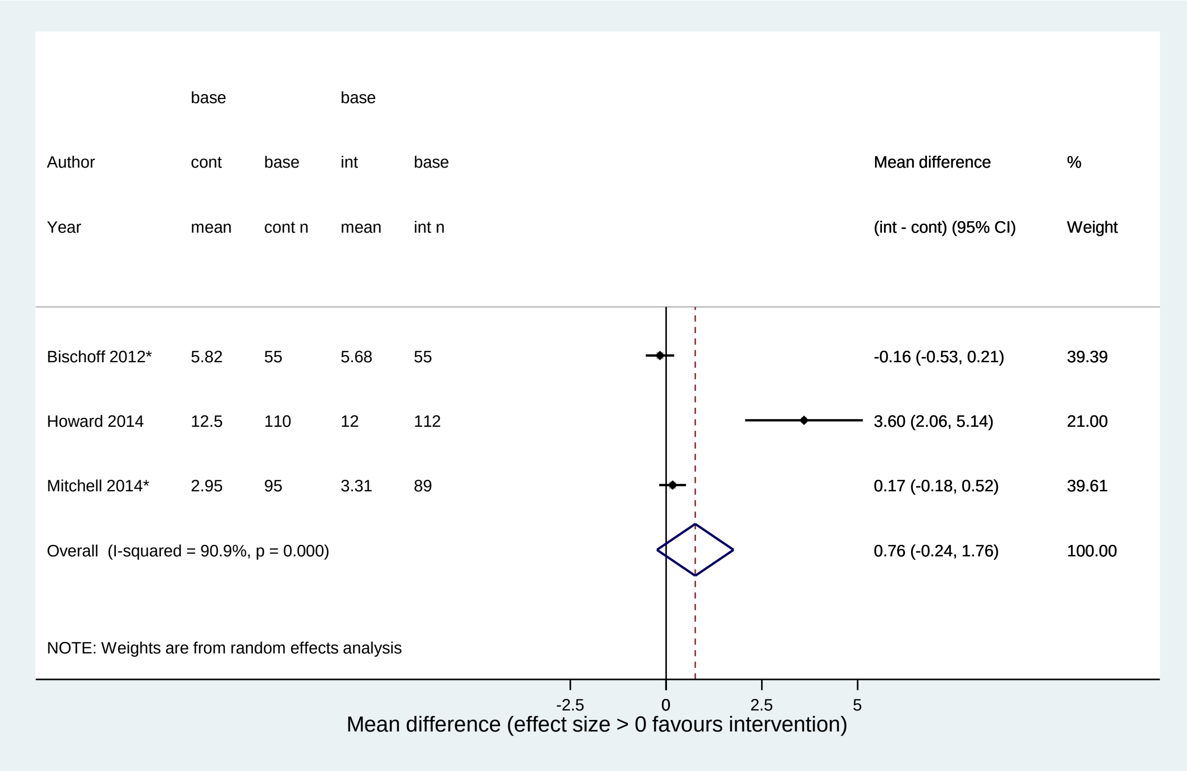

Supplementary Figure 5: Meta-analysis of CRQ-emotions scores

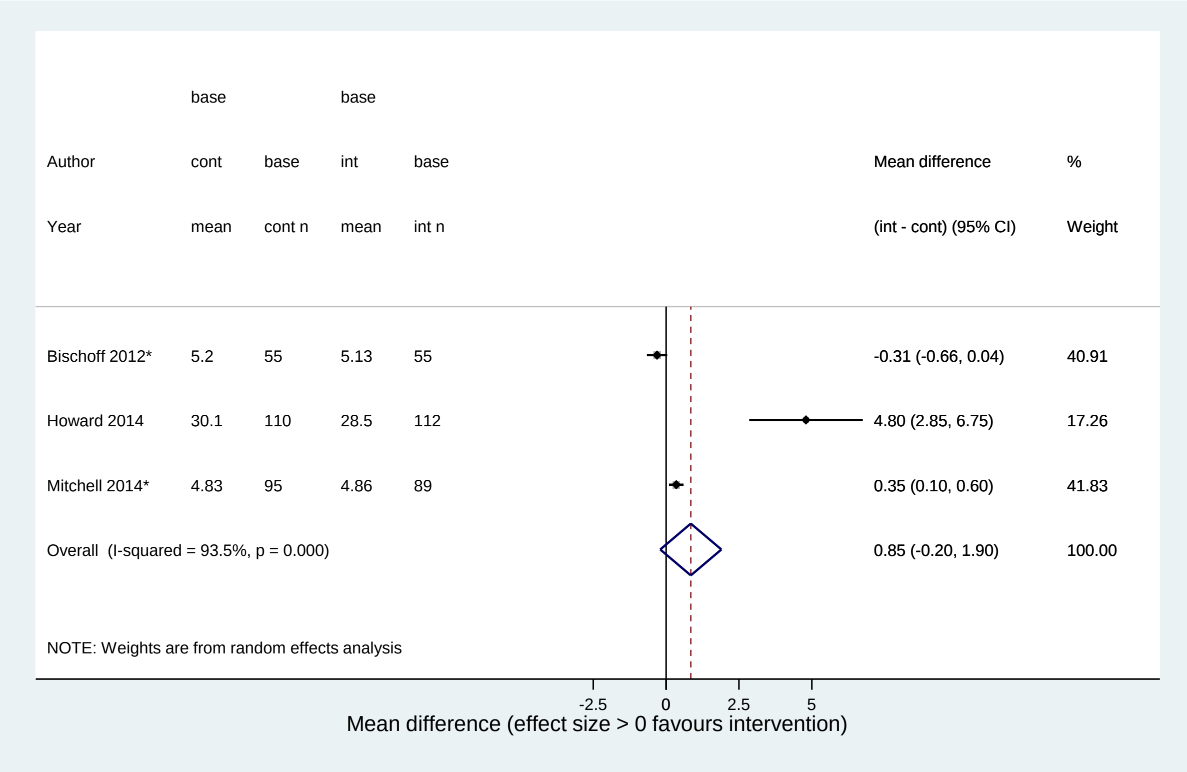

Supplementary Figure 6: Meta-analysis of CRQ-fatigue scores

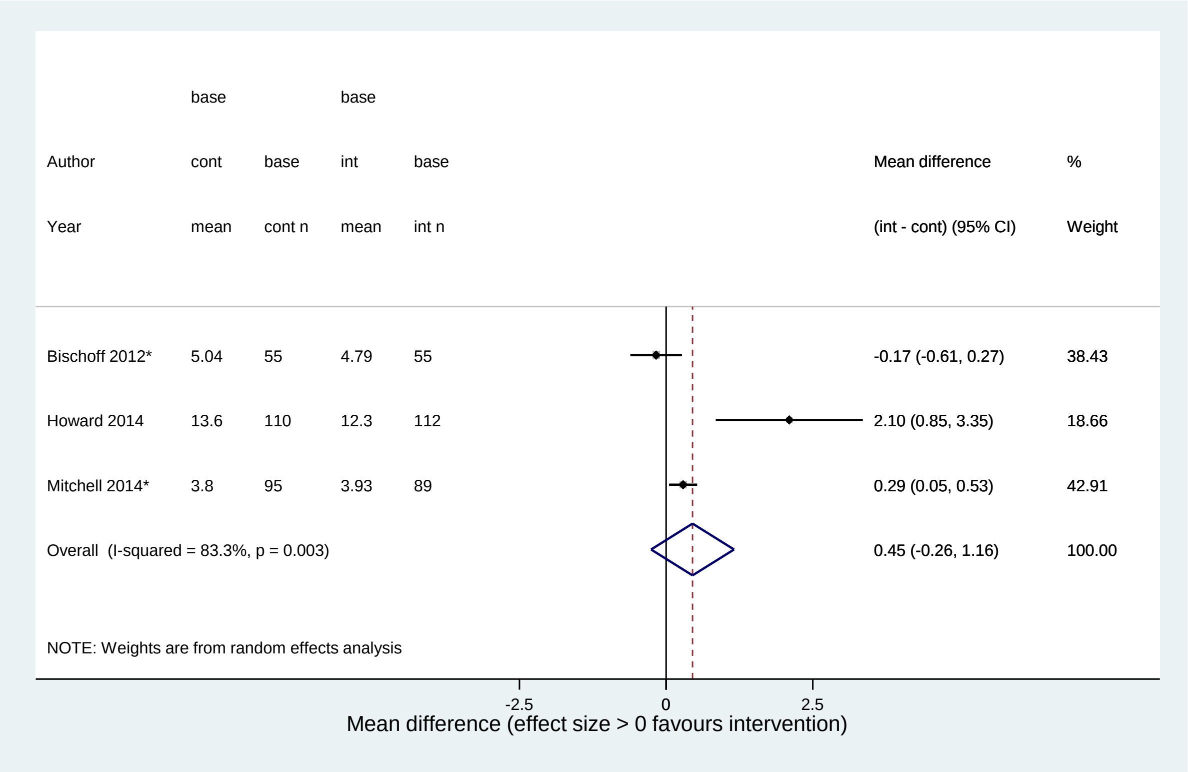

Supplementary Figure 7: Meta-analysis of CRQ-mastery scores

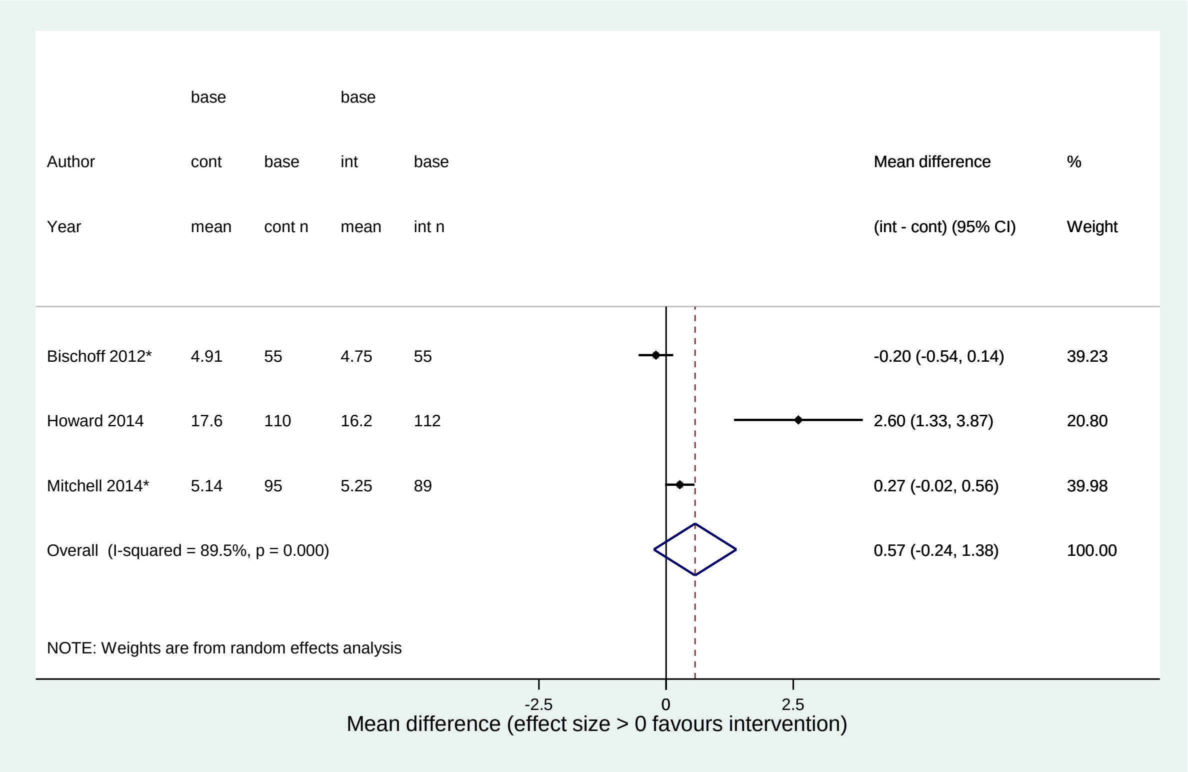

Supplementary Figure 8: Meta-analysis of HADS anxiety scores

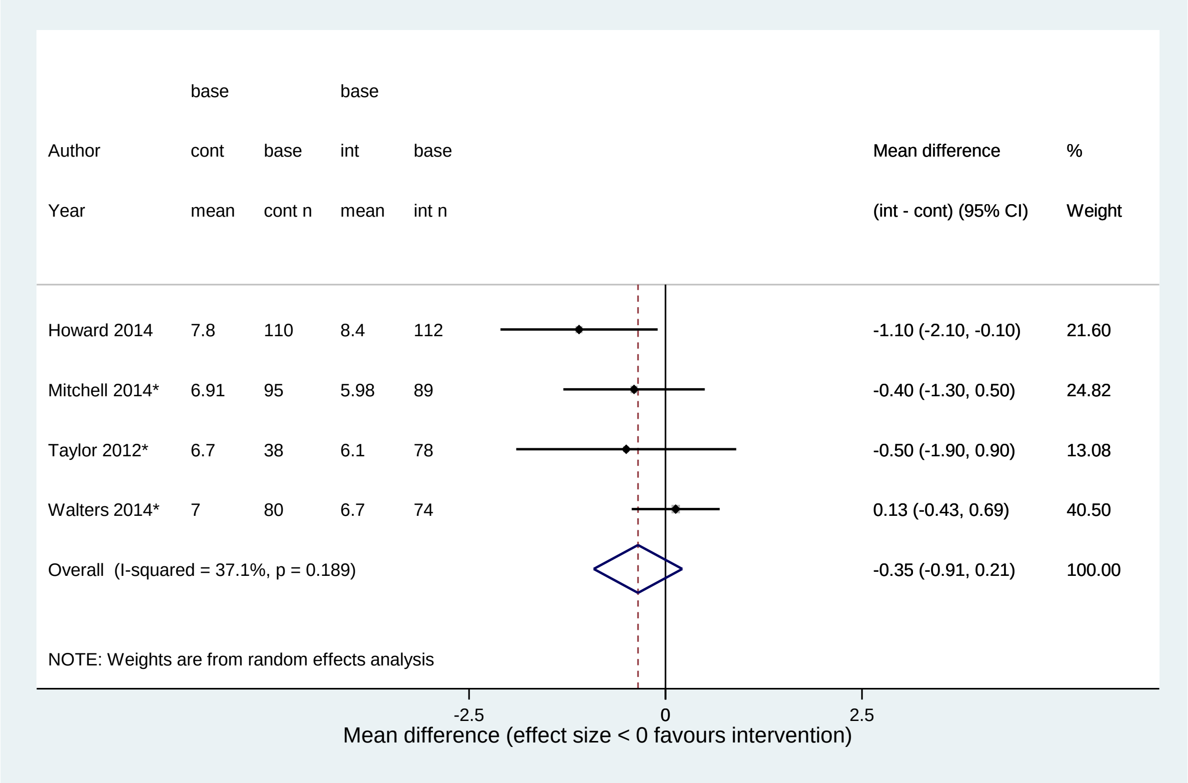

Supplementary Figure 9: Meta-analysis of HADS depression scores

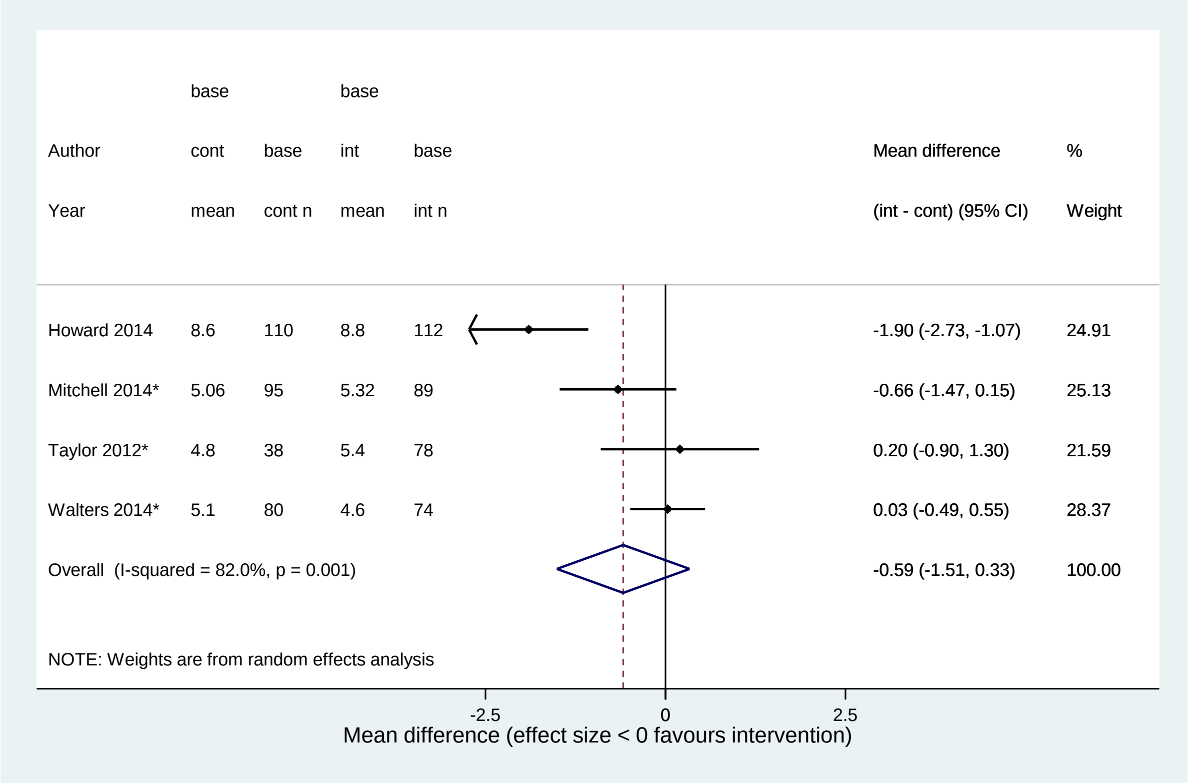

Supplementary methods – Search strategy for Medline

Database: Ovid MEDLINE(R) In-Process & Other Non-Indexed Citations October 05, 2017

Search Strategy:

1 chronic obstructive pulmonary disease.mp.

2 copd.mp.

3 chronic obstructive lung disease.mp.

4 chronic obstructive airway disease.mp.

5 chronic respiratory disorder$.ti,ab.

6 smoking-related lung disease$.ti,ab.

7 emphysema.ti,ab.

8 bronchitis.ti,ab.

9 or/1-8

10 (self adj2 (support$ or care or caring or manage$)).ti,ab.

11 post discharge.ti,ab.

12 early discharge.ti,ab.

13 home care.ti,ab.

14 home nursing.ti,ab.

15 patient centred care.ti,ab.

16 patient centered care.ti,ab.

17 patient education.mp.

18 patient participation.ti,ab.

19 post hospital care.ti,ab.

20 action planning.ti,ab.

21 discharge planning.ti,ab.

22 (continuity adj2 care).ti,ab.

23 (support$ adj2 (discharge or manage$)).ti,ab.

24 patient focus$.ti,ab.

25 management plan$.mp.

26 management program$.ti,ab.

27 rehabilitation.ti,ab.

28 or/10-27

29 9 and 28

30 community.mp.

31 family physician$.mp.

32 GP$.mp.

33 (general practitioner$ or general practice).mp. [mp=title, abstract, original title, name of substance word, subject heading word, keyword heading word, protocol supplementary concept word, rare disease supplementary concept word, unique identifier, synonyms]

34 (family practition$ or family practic$).mp. [mp=title, abstract, original title, name of substance word, subject heading word, keyword heading word, protocol supplementary concept word, rare disease supplementary concept word, unique identifier, synonyms]

35 (primary adj (care or healthcare)).ti,ab.

36 or/30-35

37 29 and 36

38 limit 37 to yr="2016 - 2017"

Database: Ovid MEDLINE(R) 1946 to September Week 4 2017

Search Strategy:

1 chronic obstructive pulmonary disease.mp. or exp Pulmonary Disease, Chronic Obstructive/

2 copd.mp.

3 chronic obstructive lung disease.mp.

4 chronic obstructive airway disease.mp.

5 chronic respiratory disorder$.mp.

6 smoking-related lung disease$.mp.

7 Pulmonary Emphysema/

8 exp Bronchitis/

9 emphysema.mp.

10 or/1-9

11 exp Self Care/

12 (self adj2 (support$ or care or caring or manage$)).mp. [mp=title, abstract, original title, name of substance word, subject heading word, keyword heading word, protocol supplementary concept word, rare disease supplementary concept word, unique identifier, synonyms]

13 post discharge.mp.

14 early discharge.mp.

15 home care.mp.

16 home care services/ or home nursing/

17 patient centred care.mp.

18 patient centered care.mp.

19 patient education/ or patient education.mp.

20 patient participation.mp.

21 post hospital care.mp.

22 action planning.mp.

23 discharge planning.mp.

24 continuity of patient care/

25 (support$ adj2 discharge).mp. [mp=title, abstract, original title, name of substance word, subject heading word, keyword heading word, protocol supplementary concept word, rare disease supplementary concept word, unique identifier, synonyms]

26 (support$ adj2 manag$).mp. [mp=title, abstract, original title, name of substance word, subject heading word, keyword heading word, protocol supplementary concept word, rare disease supplementary concept word, unique identifier, synonyms]

27 patient focus$.mp.

28 management plan$.mp.

29 management program$.mp.

30 rehabilitation.mp. or exp Rehabilitation/

31 or/11-30

32 10 and 31

33 community.mp.

34 family physician$.mp. or exp Physicians, Family/

35 GP$.mp.

36 exp Family Practice/ or exp General Practice/

37 (general practitioner$ or general practice).mp. [mp=title, abstract, original title, name of substance word, subject heading word, keyword heading word, protocol supplementary concept word, rare disease supplementary concept word, unique identifier, synonyms]

38 (family practition$ or family practic$).mp. [mp=title, abstract, original title, name of substance word, subject heading word, keyword heading word, protocol supplementary concept word, rare disease supplementary concept word, unique identifier, synonyms]

39 or/33-38

40 32 and 39

41 limit 40 to "therapy (maximizes sensitivity)"

42 limit 41 to yr="2016 - 2017"
